# Supplementary material for: Gender difference on the mediation effects of filial piety on the association between chronic obstructive pulmonary disease and depressive symptoms in older adults: A community-based study
Source: PLoS One. 2024 Feb 22;19(2):e0298360. doi: 10.1371/journal.pone.0298360 (PMC10883558; doi:10.1371/journal.pone.0298360)
Supplement: S1 Table — (DOCX) [file pone.0298360.s001.docx]

S1 Table. Comparisons of characteristics between people with non-COPD and COPD.

|  |  | Lung function | |  |
| --- | --- | --- | --- | --- |
|  | All | non-COPD | COPD^＊^ | *P* Value^◎^ |
| N | 1462 | 1100 | 362 |  |
| Age | 74.0±7.6 | 73.7±7.7 | 75.1±7.2 | 0.002 |
| ≥ 65 | 1261(86.3) | 931(84.6) | 330(91.2) | 0.002 |
| Men | 727(49.7) | 554(50.4) | 173(47.8) | 0.396 |
| BMI | 24.5±3.5 | 24.5±3.4 | 24.5±3.9 | 0.702 |
| Waist circumference | 88.6±10.1 | 88.2±9.9 | 89.7±10.9 | 0.018 |
| Marital status |  |  |  | 0.160 |
| unmarried | 27(1.9) | 22(2.0) | 5(1.4) |  |
| divorced | 61(4.2) | 45(4.1) | 16(4.4) |  |
| widowed | 401(27.5) | 286(26.1) | 115(31.9) |  |
| married | 969(66.5) | 744(67.8) | 225(62.3) |  |
| Education level |  |  |  | <.001 |
| < high school | 865(59.3) | 617(56.2) | 248(68.7) |  |
| high school | 289(19.8) | 233(21.2) | 56(15.5) |  |
| > high school current | 305(20.9) | 248(22.6) | 57(15.8) |  |
| Smoking status |  |  |  | <.001 |
| non-smokers | 1048(71.7) | 810(73.6) | 238(65.8) |  |
| ex-smokers | 283(19.4) | 210(19.1) | 73(20.2) |  |
| current-smokers | 131(9.0) | 80(7.3) | 51(14.1) |  |
| Exercise habit | 990(67.7) | 775(70.5) | 215(59.4) | <.001 |
| Past history |  |  |  |  |
| Hypertension | 770(52.7) | 559(50.8) | 211(58.3) | 0.014 |
| Diabetes mellitus | 328(22.4) | 242(22.0) | 86(23.8) | 0.487 |
| Stroke | 114(7.8) | 68(6.2) | 46(12.7) | <.001 |
| CAD | 423(28.9) | 313(28.5) | 110(30.4) | 0.482 |
| Psychiatric disease | 61(4.2) | 42(3.8) | 19(5.3) | 0.238 |
| Fasting glucose | 107.8±28.8 | 108.0±29.9 | 107.1±25.0 | 0.605 |
| Insulin resistance | 7.1±7.6 | 7.2±8.3 | 6.9±4.5 | 0.530 |
| Total cholesterol | 186.8±37.2 | 187.0±37.8 | 186.4±35.4 | 0.792 |
| HDL | 52.9±14.9 | 52.8±14.7 | 53.1±15.5 | 0.742 |
| LDL | 109.7±32.6 | 109.9±33.2 | 109.1±30.8 | 0.700 |
| TG | 125.1±76.5 | 124.3±78.9 | 127.6±68.2 | 0.484 |
| Albumin | 4.36±0.25 | 4.37±0.24 | 4.35±0.26 | 0.114 |
| Systemic inflammation |  |  |  |  |
| hsCRP | 0.24±0.54 | 0.23±0.52 | 0.28±0.58 | 0.115 |
| Interleukin-6 | 2.50±2.09 | 2.41±2.05 | 2.77±2.21 | 0.006 |
| Fibrinogen | 272.2±64.1 | 271.0±63.2 | 275.9±66.8 | 0.213 |
| D-dimer | 0.67±0.97 | 0.67±1.01 | 0.69±0.82 | 0.765 |
| MMSE | 25.4±4.2 | 25.8±3.9 | 24.2±4.9 | P<0.001 |
| Mediating factors |  |  |  |  |
| Filial Piety |  |  |  |  |
| Filial piety expectation | 18.06±5.20 | 17.92±5.13 | 18.51±5.38 | 0.078 |
| Perceived filial piety receipt | 19.90±4.87 | 19.83±4.80 | 20.12±5.09 | 0.343 |
|  |  |  |  |  |
| CES-D | 4.56±5.70 | 4.15±5.19 | 5.80±6.88 | <.001 |
| ≥ 16 | 70(4.8) | 40(3.6) | 30(8.3) | <.001 |

^＊^COPD: FEV1< 70% predicted or FEV1/ FVC< 0.7; ^◎^*P* Value: Chi-Square test、ANOVA

BMI: body mass index; CAD: coronary artery disease;

HDL: high-density lipoprotein; LDL: low-density lipoprotein; TG: triglyceride;

CES-D: Center for Epidemiologic Studies Depression Scale

MMSE: Mini-Mental State Examination
