# Supplementary material for: Gender difference on the mediation effects of filial piety on the association between chronic obstructive pulmonary disease and depressive symptoms in older adults: A community-based study
Source: PLoS One. 2024 Feb 22;19(2):e0298360. doi: 10.1371/journal.pone.0298360 (PMC10883558; doi:10.1371/journal.pone.0298360)
Supplement: Attachment — Submitted filename: PlosOne-reviewer2023.docx [file pone.0298360.s002.docx]

Dear Editor:

Thanks for giving us the opportunity to revise the manuscript. We hope this version meets your standard. We have revised the manuscript according to the requirements of editorial office and the comments of reviewers. Following is the point-by point response to reviewers. We added a supplementary table for comparing those with COPD to those without. Some coefficients changed a little after MMSE was adjusted in the model. The conclusion remained the same.

Part of this study was sponsored by the National Health Research Institutes (PH-110-SP-01). The funding is mainly for data collection. There was no additional external funding received for this study. The funders had no role in study design, data collection and analysis, decision to publish, or preparation of the manuscript.

Regards,

Hsing-Yi Chang

Reviewer #1: It is a very interesting study and thank you for allowing me to review this particular research article. Agreed that perhaps more studies are required to find the association between filial piety and depression with worsening lung functions in COPD patients. Future studies may be needed to explore this gap between filial piety, FPR and their outcome thereof in COPD patients. There are a lot of psychosocial factors responsible for depression. Other than that there are also some biological factors which may account for depression like dementia, vascular factors, medical comorbidities etc. Were these taken into account as these could affect the outcome of the study?

**Reply:** Thanks for reminding us the other factors associated with depression. We compared the COPD with non-COPD and found that those with COPD had higher proportion of chronic diseases (supplementary Table 1) and lower cognitive function (MMSE). The inflammatory factors were not different between groups. Most of the participants were able to answer the questions. Those with sever dementia were excluded from the interview. We used MMSE to evaluate their cognitive function (supplementary Table 1). They were different between people with COPD and without COPD. However, their scores indicated they were in relatively good cognitive function. The final model adjusted for age, waist, education, smoking status, hypertension, stroke and MMSE. We did not adjust MMSE in previous manuscript. It was added to the model and did not alter the conclusion. Some coefficients were changed, but the relationships remained the same. We revised the coefficients accordingly. We added one sentence in the section of material and method ‘Age, waist, education, smoking status, hypertension, stroke, and MMSE were adjusted in the final model.’ (Lines 169-170). The footnote of related tables also indicated that these variables were adjusted in the model.

Supplementary Table 1. Comparisons of characteristics between people with non-COPD and COPD.

|  |  | Lung function | |  |
| --- | --- | --- | --- | --- |
|  | All | non-COPD | COPD^＊^ | *P* Value^◎^ |
| N | 1462 | 1100 | 362 |  |
| Age | 74.0±7.6 | 73.7±7.7 | 75.1±7.2 | 0.002 |
| ≥ 65 | 1261(86.3) | 931(84.6) | 330(91.2) | 0.002 |
| Men | 727(49.7) | 554(50.4) | 173(47.8) | 0.396 |
| BMI | 24.5±3.5 | 24.5±3.4 | 24.5±3.9 | 0.702 |
| Waist circumference | 88.6±10.1 | 88.2±9.9 | 89.7±10.9 | 0.018 |
| Marital status |  |  |  | 0.160 |
| unmarried | 27(1.9) | 22(2.0) | 5(1.4) |  |
| divorced | 61(4.2) | 45(4.1) | 16(4.4) |  |
| widowed | 401(27.5) | 286(26.1) | 115(31.9) |  |
| married | 969(66.5) | 744(67.8) | 225(62.3) |  |
| Education level |  |  |  | <.001 |
| < high school | 865(59.3) | 617(56.2) | 248(68.7) |  |
| high school | 289(19.8) | 233(21.2) | 56(15.5) |  |
| > high school current | 305(20.9) | 248(22.6) | 57(15.8) |  |
| Smoking status |  |  |  | <.001 |
| non-smokers | 1048(71.7) | 810(73.6) | 238(65.8) |  |
| ex-smokers | 283(19.4) | 210(19.1) | 73(20.2) |  |
| current-smokers | 131(9.0) | 80(7.3) | 51(14.1) |  |
| Exercise habit | 990(67.7) | 775(70.5) | 215(59.4) | <.001 |
| Past history |  |  |  |  |
| Hypertension | 770(52.7) | 559(50.8) | 211(58.3) | 0.014 |
| Diabetes mellitus | 328(22.4) | 242(22.0) | 86(23.8) | 0.487 |
| Stroke | 114(7.8) | 68(6.2) | 46(12.7) | <.001 |
| CAD | 423(28.9) | 313(28.5) | 110(30.4) | 0.482 |
| Psychiatric disease | 61(4.2) | 42(3.8) | 19(5.3) | 0.238 |
| Fasting glucose | 107.8±28.8 | 108.0±29.9 | 107.1±25.0 | 0.605 |
| Insulin resistance | 7.1±7.6 | 7.2±8.3 | 6.9±4.5 | 0.530 |
| Total cholesterol | 186.8±37.2 | 187.0±37.8 | 186.4±35.4 | 0.792 |
| HDL | 52.9±14.9 | 52.8±14.7 | 53.1±15.5 | 0.742 |
| LDL | 109.7±32.6 | 109.9±33.2 | 109.1±30.8 | 0.700 |
| TG | 125.1±76.5 | 124.3±78.9 | 127.6±68.2 | 0.484 |
| Albumin | 4.36±0.25 | 4.37±0.24 | 4.35±0.26 | 0.114 |
| Systemic inflammation |  |  |  |  |
| hsCRP | 0.24±0.54 | 0.23±0.52 | 0.28±0.58 | 0.115 |
| Interleukin-6 | 2.50±2.09 | 2.41±2.05 | 2.77±2.21 | 0.006 |
| Fibrinogen | 272.2±64.1 | 271.0±63.2 | 275.9±66.8 | 0.213 |
| D-dimer | 0.67±0.97 | 0.67±1.01 | 0.69±0.82 | 0.765 |
| MMSE | 25.4±4.2 | 25.8±3.9 | 24.2±4.9 | P<0.001 |
| Mediating factors |  |  |  |  |
| Filial Piety |  |  |  |  |
| Filial piety expectation | 18.06±5.20 | 17.92±5.13 | 18.51±5.38 | 0.078 |
| Perceived filial piety receipt | 19.90±4.87 | 19.83±4.80 | 20.12±5.09 | 0.343 |
|  |  |  |  |  |
| CES-D | 4.56±5.70 | 4.15±5.19 | 5.80±6.88 | <.001 |
| ≥ 16 | 70(4.8) | 40(3.6) | 30(8.3) | <.001 |

^＊^COPD: FEV1< 70% predicted or FEV1/ FVC< 0.7; ^◎^*P* Value: Chi-Square test、ANOVA

BMI: body mass index; CAD: coronary artery disease;

HDL: high-density lipoprotein; LDL: low-density lipoprotein; TG: triglyceride;

CES-D: Center for Epidemiologic Studies Depression Scale

MMSE: Mini-Mental State Examination

Other factors which probably could play a very important role in the study would be the education status of the individual, annual income, marital status, living arrangements, comorbidities of the spouse, immigration status. Could you please throw some light on these factors?

**Reply**: As shown in supplementary Table 1, education was significantly different between the non-COPD and COPD groups, whereas the marital status was not. Education level was adjusted in the final model. We did not have information about the comorbidities of the spouse. Immigration status might not as important as in the US, since most of the participants were Chinese living in Taiwan for long-time.

Regarding the COPD status of the individuals, how many of them were on LTOT, home BiPAP or CPAP therapy possibly reflecting the more severe status of the disease and thereby assuming that the level of anxiety and depression in these patients would be more? Was there any such correlation?

Thank you and awaiting your response.

**Reply**: We did not have the information about the therapy of the COPD patients. We modeled the lung function as a continuous variable, which might catch various status of patients.

Reviewer #2: This is a very interesting paper.

Was there a review of the population's history of mental illness or use of antidepressant treatment? since the patient with mental illness is known to smoke more.

**Reply:** We only used the questionnaire data for the analysis. There was no information on the use of antidepressant. These were community dwelling older adults. However, smoking status was controlled in the model.

These patients have a Gold classification for their COPD, since the greater the severity of the disease a greater degree of depression they may have.

**Reply:** Indeed, the COPD status might affect the depression status. We analyzed the data using both lung function and depressive symptoms as continuous variables instead of categorical variables. That might reflect the relationship between increasing lung function and depressive symptoms.
